# The Book World of Medicine and Science

**Published:** 1893-03-25

**Authors:** 


### The Book World of Medicine and Science.

### FIRST IMPRESSIONS OF BOOKS RECEIVED.

[It is requested that all books intended to be noticed in this column may be sent to the Editor, at the Office, 428, Strand, W.C., not later than Tuesday evening in each week.]

The Executive Council of the Imperial Institute have issued, in pamphlet form, an account of the origin and development of the Institute, and a statement of its objects and purposes. To this is added a description of the site and the buildings, with several illustrations of the hall, corridors, staircases, &c. The details concerning the Fellowship of the Institute are also given, and the pamphlet gives just the information required by those who are interested in or want to join the Institute.

Notes on Medicinal Remedies. By J. B. Stephenson, Member of the Pharmaceutical Society of Great Britain, ex-President of the British Pharmaceutical Conference. (London: Baillière, Tindall, and Co.)

A small book written for intelligent laymen, consisting of very brief descriptions of some of the best-known drugs and their principal uses. There is no attempt at originality. The book may interest laymen, but it will prove a dangerous substitute for a medical man's advice.

Cheyne-Stokes Respiration. By George Alexander Gibson, M.D., D.Sc., F.R.C.P.E., F.R.S.E., Assistant Physician to the Royal Infirmary of Edinburgh, &c. (Edinburgh: Oliver and Boyd. 1892.)

An elaborate essay on this interesting phenomenon. Much of it consists of statements of the views of other writers of all countries, and is the outcome of wide and careful reading. We have also the clinical records of six cases observed by the author, and some interesting critical remarks. Dr. Gibson views this form of respiration as one of a series of associated symptoms, and he attributes the periodic variation in the functional activity of the automatic centre for respiration to the loss of influence of the higher nervous centres, or to the irregular discharge of unequal impulses from these centres.

Authority in Medicine and its Limits. By James F. Goodhart, M.D., F.R.C.P., Physician to Guy's Hospital. (Birmingham: Hall and English.)

Dr. Goodhart delivered the inaugural address of the Birmingham and Midland Medical Society in November last. It was published in the *Birmingham Medical Review* for December, and is now reprinted. All that Dr. Goodhart writes is well worth reading. He is a genial philosopher, a kindly critic, a wise cynic, who has the knack of blending wisdom with merriment, and charms all his readers.

L'Assistance. Bulletin Officiel de la Policlinique de Paris. Third année. No. 4.

This number contains a report by M. Puteaux, the Vice-President of the Polyclinic, on the proposal to have paying consultations in addition to the free ones which have been hitherto exclusively given. The decision is given against the innovation, and a threatened financial difficulty has been got over by some economies, and especially by an extra grant of money from the Municipal Council of Paris and the General Council of the Seine.

Tuberculosis of Bones and Joints. By N. Senn, M.D., Ph.D. (Philadelphia and London: F. A. Davis and Co.)

Dr. Senn is a prolific writer, and in this volume has attacked one phase of the disease which is the most important

THE BOOK WORLD OF MEDICINE AND SCIENCE.—(*continued*).

of all to which humanity is subject. His method is to quote largely from all the most recent writers and investigators on the subject, and present his readers with the latest results. This plan has its advantages for the few, but the many require their pabulum more condensed and ready digested. It is a capital book.

### HOSPITAL BYE-LAWS.

The Rules, Orders, and Charges of St. Thomas's Hospital have recently been thoroughly revised. They extend to great length, and the new edition is a book of 248 pages, demi octavo. The new rules and orders came into operation and took effect from January 1st last. As the Lords' Committee pointed out, St. Thomas's Hospital assigns a larger share in the administration to itself than either Bart's or Guy's; still, in practice, as these new rules show, the governors have little voice in the management of the affairs and estates of this great Corporation. In our view, and in the view of many of the governors, this is a misfortune, and we look forward to the day when, under the auspices and at the instigation of the present able treasurer, Mr. Wainwright, the governors of St. Thomas's Hospital will be encouraged and enabled to take an active and continuous part in the administration of its affairs.

### POOR LAW REFORM.

The Poor Law Reform Association, of which Mr. Montmorency, Hyde Vale, Greenwich, is the honorary secretary, has issued a most useful list of questions for candidates in connection with the approaching elections of guardians of the board. Anyone interested may obtain a copy of these questions by writing to Mr. Montmorency. The subjects dealt with cover the proper clothing of the girls in workhouses and district schools, and the placing them under the direct supervision of the members of the boards; the boarding-out of pauper children; the dressing of the children in some material that will not stigmatise them as paupers, and so help to dissociate them from the workhouse; the nursing of sick paupers by trained nurses, as opposed to incompetent paupers; provision of separate apartments for married people; the provision, through Lady Meath, 83, Lancaster Gate, W., of books, newspapers, spectacles, and interesting occupations for the aged poor; the alleviation of labour in the case of casuals; provision for the aged poor to go out every fine day, and an allowance of tobacco or snuff; and the placing of the infants and children under the care of suitable attendants, in contradistinction to pauper women who are mentally and morally unfit to undertake their custody. These questions cover a wide field, and their suggestive character ought to prove almost an education to many candidates who aspire to fill the office of guardian of the poor.

